# 体外循环在普胸外科手术的应用和前景

**DOI:** 10.3779/j.issn.1009-3419.2018.04.18

**Published:** 2018-04-20

**Authors:** 海涛 马

**Affiliations:** 苏州大学附属第一医院胸外科 Department of Thoracic Surgery, First Affiliated Hospital to Suzhou University

局部晚期肿瘤的治疗一直是胸外科领域的难点，尤其是累及心脏或大血管的肿瘤，手术风险高，难度大，围手术期并发症发生率高，对于手术指征和手术方式的选择一直存在较多争议，对于常规手术方式无法切除或者手术风险极大的局部晚期肿瘤，体外循环增加手术切除的机会，但也会增加术后并发症的发生率，目前该项技术在普胸外科的应用仍有许多有待解决的问题。

本文作者探讨了在体外循环辅助下的累及心脏或大血管的胸部肿瘤的外科治疗，回顾性分析了11例患者接受体外循环辅助下胸部肿瘤切除手术的疗效，其中肺恶性肿瘤2例，纵隔肿瘤9例。术后病理结果：肺鳞癌1例，肺平滑肌肉瘤1例，心包腔内纤维肉瘤1例，侵袭性纤维瘤1例，胸腺瘤4例，胸腺癌2例，淋巴瘤1例，所有肿瘤均累及心脏或大血管。其中淋巴瘤属于血液系统肿瘤，如果不是压迫重要器官而危及生命或者非手术切除不能确诊，手术指征需要严格把握。心包腔纤维肉瘤和纵隔侵袭性纤维瘤发病率极低，对于这类肿瘤的治疗方式尚无定论。

对于胸腺瘤和胸腺癌患者，目前的研究均提示肿瘤完整切除与预后密切相关，完全切除的胸腺癌患者预后优于未手术者，完整切除肿瘤是其重要治疗方式，因此对于累及心脏大血管的胸腺瘤或胸腺癌患者，采用在体外循环辅助下完整切除的手术方法，可能是值得尝试的方式，但是由于相关病例较少，尚缺乏足够的循证学依据，而且此类前瞻性研究开展的难度也比较大。

本研究报道2例肺恶性肿瘤在体外循序下行手术切除的病例，累及心脏或大血管的T4期肿瘤传统上被视为手术禁忌，但近年有研究^[1, 2]^报道扩大切除能够达到局部根治，术后远期疗效优于单纯保守治疗，国内周清华教授等^[3]^报道349例侵犯心脏大血管的非小细胞肺癌的治疗结果，手术死亡率为0.6%，术后五年生存率为33%，明显优于保守治疗。国外同行在这一领域也进行了大胆的尝试，Muralidaran等^[4]^对近20年内非小细胞中心型肺癌于体外循环下行左肺全切除术后相关文献进行了荟萃分析，发现5年存活率达37%（中位生存率达3年）。近年来，国内亦有报道使用体外膜肺氧合（extracorporeal membrane oxygenation, ECMO）辅助下行进肺癌手术，在这个领域进行探索。

在体外循环情况下，病人血流动力学稳定，心肺均处于无血状态，大血管张力降低，手术可以在无血条件下完整切除，使得大出血可能降低，且一旦发生出血，也可以从容的修补止血，提高手术切除率和安全性。

体外循环用于肺癌手术的优点：

1.在体外循环下行肺癌根治术，肺和心脏均无血流，可在无血流条件下对侵犯心脏、大血管的晚期肺癌进行切除；可以进行气体交换、提供无血手术野，可大大降低外科手术的危险性；体外循环可以调节温度，通过其加热功能，可能对肿瘤产生疗效。

2.手术过程中无需换气，术者可将气管、支气管任意开放，为施行较复杂的气管、隆凸或支气管重建提供便利。

3.术中可避免手术钳阻断下的操作，从而减少剥离和压迫肿瘤，降低术中肿瘤播散的机会。

体外循环用于肺癌手术的缺陷：

1.技术性：目前国内各大医院已陆续将胸外科和心脏大血管外科分离，形成相对独立的科室，专业更加细化，同时带来的问题是许多胸外科医师对心脏大血管领域相关技术不了解，特别是体外循环的流程和技巧不熟悉，对手术的实施造成一定的困难。这就要求胸外科医师，特别是年轻医师平时应注意跨学科和专业的学习，不能固守自家的一亩三分地。

2.创伤性：体外循环具有一定的创伤性，如缺血再灌注损伤，中枢神经损伤和血液破坏等；是否会增加肿瘤血行播散的可能性，从而影响其预后仍需深入研究。

3.费用：体外循环相关手术耗材的使用必然会带来手术费用的增加，加重患者和医保的经济负担，这也是术者应考虑的问题。

总的来讲，尽管存在一些复杂的问题，对于累及心脏或大血管的局部晚期肿瘤，体外循环下的手术切除仍然是一种值得探索和研究的技术选择。
